# Thermal Imaging for Teaching Materials Chemistry: Phase Change Materials toward Energy Transition

**DOI:** 10.1021/acs.jchemed.5c00079

**Published:** 2025-06-10

**Authors:** Carmen de Cabo-Rodríguez, Cayetana Torremocha-Serra, Ángel Ferradanes-Martínez, María Gelpi, Pedro Dafonte-Rodríguez, Lorena Alonso-Marañón, Socorro Castro-García, Juan Manuel Bermúdez-García

**Affiliations:** CICA−Centro Interdisciplinar de Química e Bioloxía e Departamento Química, Facultade de Ciencias, Universidade da Coruña, 15071 A Coruña, Spain

**Keywords:** General, Laboratory Instructions, Multidisciplinary, Hands-on Learning, Discovery Learning, Materials Science, Phase Transitions, Applications of Chemistry

## Abstract

Nowadays, materials for energy transition is a key topic in many academic programs in chemistry, natural sciences, and engineering at different levels, especially in advanced undergraduate and postgraduate courses. In this regard, one of the emerging topics is eco-friendly refrigeration and heating solutions based on phase change materials (PCMs). Nevertheless, recent studies show that senior high school and junior university students still find it difficult to relate these phase changes with chemistry. In this work, we have designed visually appealing experiments that use thermal cameras (increasingly affordable) and inexpensive materials, focusing on the physicochemistry of phase transitions and some of their innovative applications.

## Graphical abstract

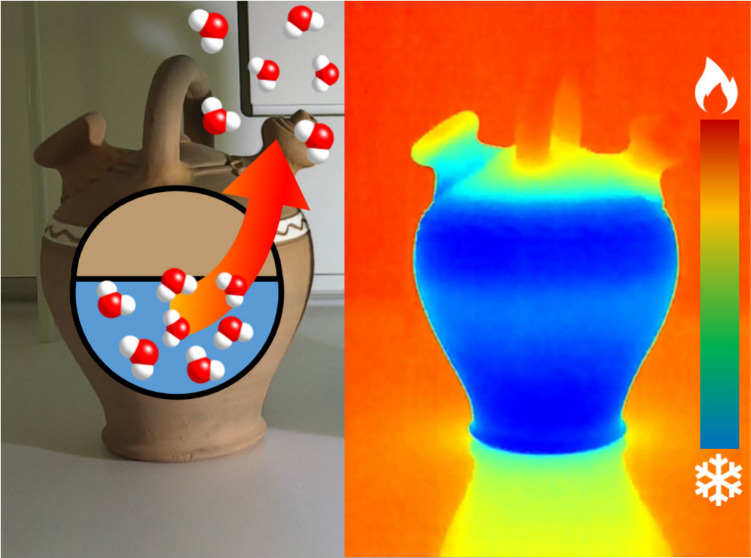

## Introduction

Phase change materials (PCMs) and their phase diagrams are fundamental cornerstones in the chemical education curriculum, often being addressed from different perspectives and at different complexity levels in subjects ranging from basic chemistry and physics to advanced inorganic chemistry, physical chemistry, and materials chemistry. Traditional learning methods—lectures where students are passive listeners—generally offer limited engagement and interaction for the students, which often leads to difficulty in understanding concepts and/or to considering chemistry a set of disconnected topics without relation within them or with real world-applications.^1–3^ These difficulties have also been observed in the study of phase changes within both senior high school students and junior university students.^4,5^ For those reasons, innovative methods and activities have been developed to improve the learning experience of chemistry students.^1–3^ In the particular topic of phase changes, these activities include cooperative learning through the jigsaw method,^6^ experiments on the enthalpy of crystallization with coffee mugs,^7^ or the compilation of high-pressure phase diagrams of daily life substances,^8^ among others.

Nowadays, PCMs are gaining special interest in the scenario of energy transition, especially in the field of thermal energy materials for eco-friendly heating and cooling. Phase transitions are always associated with exothermic and endothermic processes that can give rise to innovative solutions based on evaporative cooling in porous materials, thermal energy storage, or passive cooling, among others.

In the recent years, universities all around the world have demonstrated increasing interest in developing advanced educational programs (including postgraduate courses) focused on energy transition, where chemistry-based technologies and materials for energy storage are considered key subjects.^9–11^

In this scenario, we present a series of hands-on and visually appealing experiments specifically designed to maximize interest and facilitate the understanding of PCMs, essentially from a chemical point of view, with a focus on thermal energy-related applications. Here we use thermal cameras, which in the last years have emerged as innovative tools for science education for students from primary school^12–14^ to high school and university degrees^15–17^ and even for general society in public media.^18^ These devices are becoming cheaper and more accessible, and soon they will be available in any education institution.^19^ Therefore, it is time, and our objective, to start expanding the portfolio of experiments that will help better the understanding of chemical concepts. The experiments compiled in this work are mainly oriented toward advanced undergraduate chemistry courses (third and fourth academic years) and postgraduate studies on materials for the energy transition, although they can also be adapted for technical education, high school, and science communication events.

## Materials and Equipment

### Thermal Cameras

In the designed experiments, we have used two types of thermal cameras: FLIR One Pro and HIKMICRO SP40.

The FLIR One Pro is a plug-and-play thermal camera that can be connected to an smartphone. This device presents an affordable economic cost (around $500), a large recording temperature range (−20 °C to +400 °C), an accuracy of ±3–5%, and a thermal image resolution of 160 × 120. This camera can be used for qualitative and semiquantitative laboratory experiments.

The HIKMICRO SP40 is a more advanced camera with a higher cost (around $8000) but also with an integrated display screen, a broader recording temperature range (from −20 to +650 °C), and higher accuracy (±2%) and thermal resolution (480 × 360). This device can also record radiometric thermal videos to export temperature data in graphics. Therefore, it is best suited for quantitative laboratory experiments and for creating high-resolution images/videos for teaching and science communication.

### Optical Lenses

Lenses of different materials (sapphire or α-Al_2_O_3_, CaF_2_, ZnSe, GaAs, and Ge) with 20 mm diameter and 2 mm thickness were purchased from Cloudray Laser Solutions and from Ideal Vacuum Products. Borosilicate glass was purchased from Fisher Scientific.

### Chemicals and Other Materials

Sodium acetate trihydrate, CH_3_COONa·3H_2_O, was purchased from Merck and used without further purification. Ceramic pots were purchased from a local ceramic artisan from Buño, a Galician region recognized for its ceramic tradition. Magic cleaning sponges (melamine sponges) and paraffin candles were purchased in a common supermarket.

## Hazards and Safety Precautions

For the selected lenses, avoid exposure to acids and strong bases, extreme temperatures, physical damage, and abrasion dust formation. Ge causes skin/eye irritation and is harmful if inhaled; ZnSe and GaAs are toxic if swallowed/inhaled and for aquatic life. Melamine foam and sodium acetate trihydrate are not classified as toxic compounds. Manipulate all lenses and chemical substances with protective gloves and avoid skin and eye contact, inhalation, ingestion, and release to the environment. For further details, always consult the safety data sheets. Be aware of hot surfaces, as they can produce burning damage.

## Experiments and Pedagogy

These experiments are designed as a practical laboratory lecture divided in 4 sessions of 60 min each, initially oriented toward advanced courses of materials chemistry and/or specialized courses on energy transition materials. Furthermore, all experiments can be also adapted for students in technical education, high school, early stage undergraduate courses, and even for science communication activities.

The main learning outcomes for these experiments can be summarized into: (1) learning the fundamentals of infrared thermal imaging applied to materials chemistry and to the study of phase change materials, (2) learning the fundamentals of the thermal properties of phase change materials and composites and relating them to chemistry-based solutions for the energy transition, and (3) acquiring hands-on training on thermal imaging for recording, processing, and discussing qualitative and quantitative data of phase change materials. Further pedagogy details and preliminary student assessments can be found in the Supporting Information (SI).

### Experiment 1: Understanding the Basics of Thermal Imaging

This experiment is an introductory activity to become familiar with the operation of the thermal cameras and to understand the basic principles of thermography and of the emission and transmission of infrared (IR) thermal radiation (more information is available in SI).

Thermal cameras can measure the electromagnetic radiation emitted by the surface of the objects in the IR region (in the wavelength range of ∼7000–15,000 nm) and transform it into temperature measurements following the principle of blackbody radiation. Accordingly, they can produce images of temperature distribution, named thermograms, displaying a color-coded scale that can be represented in different color palettes for better contrast and detail visualization. In addition, these cameras generally come with a secondary photographic camera so they can simultaneously record thermal images (from infrared radiation) and optical images (from visible light).

Differently from traditional photographic cameras, thermal cameras measure radiation emitted with longer wavelengths than visible light and generally invisible to the human eye. In turn, with these thermal devices, we can “see” a part of the electromagnetic spectrum that our naked eyes cannot. Nevertheless, a very common mistake when first using a thermal camera is to assume that thermal cameras can also see through all objects, including walls, as misrepresented in movies.

This first experiment is designed to set the basic understanding of thermal cameras and the interaction of thermal radiation (emission, absorption, and transmission) with different materials. This experiment can be carried out with the more simple FLIR One Pro thermal camera for a starting training with the basic features of a thermal camera (visual interface, temperature readings and mapping, color palettes, etc.) before using a more advanced camera in later experiments.

In this experiment, students observe a hot object (a flask filled with hot water in our experiment) with the thermal camera, simultaneously recording the optical images and the corresponding thermal images while analyzing the difference between both images. In this way, they can see how the IR radiation emitted by the surface of the materials is clearly different depending on the temperature.

They then repeat the experiment by placing lenses made of different materials—borosilicate glass, sapphire (α-Al_2_O_3_), calcium fluoride (CaF_2_), zinc selenide (ZnSe), gallium arsenide (GaAs), and germanium (Ge)—between the camera and the observed object (Figure 1) in order to compare the transmission of the optical and the thermal IR radiation through the different compounds.

**Figure 1:**
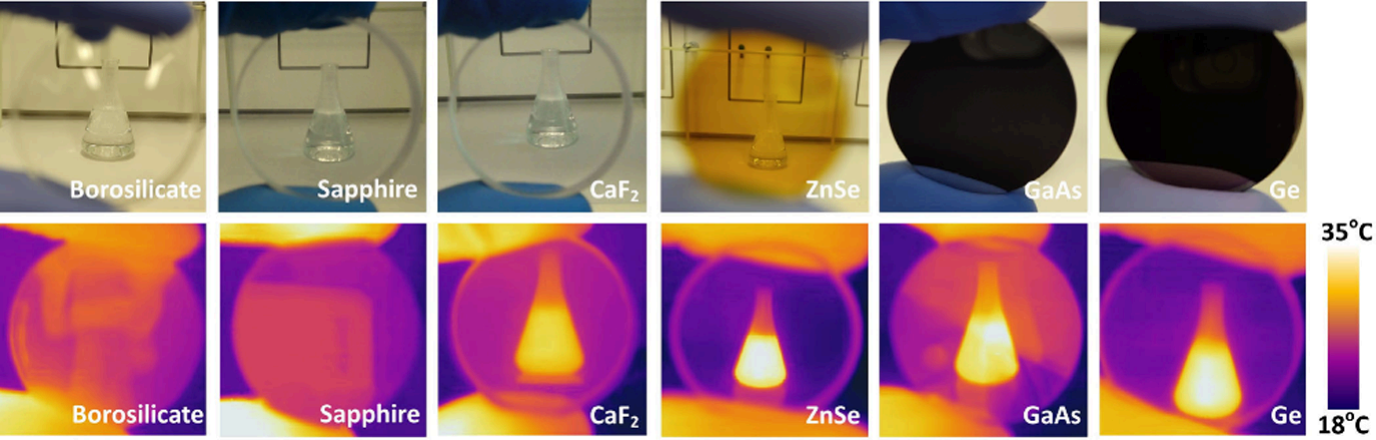
Optical images (top) and thermal images (bottom) of a hot object, taken with a FLIR One Pro thermal camera by placing different lenses between the object and the camera. Note: temperature scale represented in iron palette from yellow (hotter) to purple (colder).

As shown in Figure 1, borosilicate, sapphire, CaF_2_, and ZnSe lenses are transparent to visible light. Surprisingly, while borosilicate glass and sapphire are opaque to the IR heat radiation, CaF_2_ and ZnSe are transparent to it. On the other hand, GaAs and Ge are fully opaque to visible light while being transparent to IR heat radiation.

Senior undergraduate or postgraduate students can rationalize these observations based on their background in spectroscopy and optical properties of materials. For that purpose, they are provided with transmission spectra of the different lenses’ materials (Figure 2) so they can explain the experimental observations in terms of the electronic band structure and the lattice vibrations of the materials (see SI for further pedagogical details and questions).

**Figure 2:**
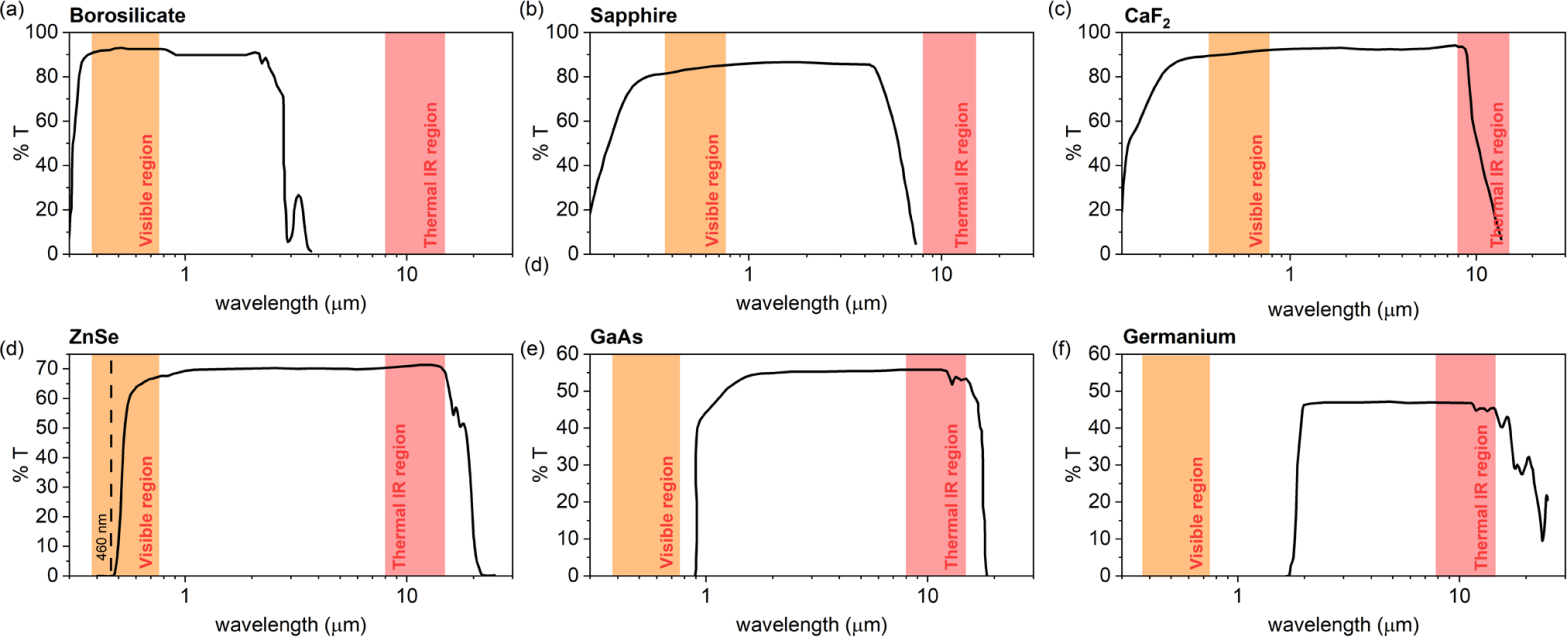
Transmission spectra from the UV–visible region up to the infrared region for the different lens materials. Data reproduced from refs 21, 25, 26 .

Using electronic band theory, photons of the visible radiation can be absorbed by electrons of the valence band and be promoted to the conduction band in a given material, leading to an optical transition. The band gap (*E*_g_) in the borosilicate glass, sapphire, and CaF_2_ is large enough, so no photon of visible light can be absorbed and, in turn, all visible light is transmitted through these lenses (Figure 2a–c).^20,21^ Meanwhile, zinc selenide (ZnSe) is a semiconductor with a large band gap that falls in the upper region of the visible range (*E*_g_ = 2.7 eV),^22,23^ absorbing photons with wavelengths lower than 460 nm or 0.46 μm (Figure 2d) and being partially transparent to the visible light. In the same line, gallium arsenide (GaAs) and germanium (Ge) are also semiconducting materials with rather smaller band gaps (*E*_g_(GaAs) = 1.42 eV; *E*_g_(Ge)=0.66 eV),^24^ absorbing photons with wavelengths lower than 870 and 1880 nm, respectively, including the whole visible rage (see Figures 2e and f).

Regarding the transmission spectrum of the thermal IR region, the borosilicate glass shows absorption for wavelengths larger than 2.5 μm related to the vibrations of the different bonds (i.e., [BO_3_], [BO_4_], [Si–O–Si], and [Si–O–B]),^27^ which hinder the transmission of thermal IR radiation (Figure 2a). Similarly, in the sapphire lens, the α-Al_2_O_3_ lattice vibrations show a large absorption in the thermal IR region with a cutoff at ∼7 μm (Figure 2b).^21^ Meanwhile, in the CaF_2_ lens, the absorption of the lattice vibrations partially lies on the thermal IR region, so the material is partially transparent to this radiation (Figure 2c).^21^ In the case of ZnSe and GaAs, the lattice vibrations are out of the thermal IR region, showing IR cutoffs at ∼25 and ∼18 μm, respectively (Figure 2d and e).^21,28^ In the same line, Ge exhibits absorption bands that are shifted to longer wavelengths above 15 μm (Figure 2f).^21^

### Experiment 2: Liquid-to-Gas Phase Transitions for Evaporative Cooling

This is a simple experiment that helps students to visualize and understand the concept of latent heat (defined as the total thermal energy that a given material can absorb/release during a phase transition) and the principle of the evaporative passive cooling.

Evaporative cooling is a refrigeration mechanism that functions through the evaporation of a liquid (generally water) by absorbing thermal energy from the environment and cooling the surroundings. This phenomenon can be easily observed in clay pots, which are traditionally used for self-refrigerating drinking water in dry regions (Figure 3). Ceramic pots for water refrigeration are made of porous clay (mainly aluminum silicate) that exhibits pores size in the submicromter range, depending on the manufacturing and sintering method.^29–31^ The water inside the pot diffuses to the surface by capillarity, where it evaporates (liquid to gas phase transition), decreasing the system temperature due to the evaporation latent heat.^32^ This is a passive cooling mechanism, which means that it does not require a connection to the electric grid or any external power source. This mechanism was widely used in entire ancient cities, such as the Hispano-Muslim Palace of Alhambra,^33^ where water evaporation from a complex hydraulic system (of fountains, pools, and cisterns) maintained the thermal comfort of the city. Even nowadays, this strategy is being imitated in modern residential spaces, and new eco-friendly thermal systems are being created using 3D printed clay pots.^31^

**Figure 3:**
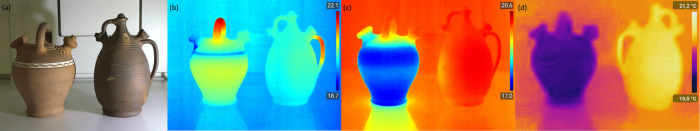
(a) Optical photographs of the ceramic pots. (b–d) Thermal images of the same pots just after water-filling the left one (b) and 1 h after water filling (c, d). Note: HIKMICRO SP40 was used to record thermal images in panels b and c in rainbow palette from red (hotter) to blue (colder); FLIR One Pro was used to record thermal image d in iron palette from yellow (hotter) to purple (colder).

Solid–liquid–gas transitions are usually explained by a prototypical diagram of the temperature evolution of a substance under heating. Here, during the phase transitions, the added thermal energy is stored in the form of latent heat (further explanations and diagrams are given in SI).

In this experiment, the students explore the thermal changes from a different perspective, observing a liquid–gas phase transition under a quasi-adiabatic environment. Under these conditions, students can observe the temperature change related to a liquid–gas phase transition. During this experiment, students monitor the temperature of two ceramic pots (one empty and the other filled with water subjected to natural evaporation) using a thermal camera. They will observe a progressive cooling of the water-filled pot that will have to be explained (Figure 3). The thermal images can be recorded using affordable smartphone thermal cameras or advanced thermal cameras for higher resolution and accuracy.

Complementary to the experimental observation, the undergraduate students can calculate the expected temperature change for a mass of water due to evaporation considering that the main contribution is the latent heat of evaporation (more details in SI). Nevertheless, the students must be aware that temperature decrease also depends on additional extrinsic factors, such as the pot size and external surface, ambient temperature, and/or humidity.^32^

### Experiment 3: Liquid-to-Solid Phase Transitions for Heating in Thermal Energy Storage Applications

This experiment is designed to illustrate the exothermic character of crystallization (that is, a liquid-to-solid phase transition) and the thermal energy storage capacity of a substance (e.g., hydrated sodium acetate). It also helps students understand concepts such as a supercooled liquid, metastable state, and mechanical activation.

Sodium acetate trihydrate (CH_3_COONa·3H_2_O), is an ideal material for thermal energy storage in domestic applications (space heating or hot water supply) due to its charging temperature when thermal energy is absorbed and stored at temperatures above the melting temperature (58 °C). Moreover, its thermal energy release can be mechanically activated at ambient (or lower) temperatures when required. Furthermore, this substance is economically accessible, nonhazardous, chemically stable, and abundant. For those reasons, hydrated sodium acetate has been largely studied (even nowadays) for eco-friendly thermal applications toward heating decarbonization.^34–36^

In the experiment, sodium acetate trihydrate is mixed with additional water to reach a mass ratio of 58 wt % CH_3_COONa and 42 wt % H_2_O.^37^ The mixture is heated above the melting temperature (*T*_m_ ∼ 58 °C) and slowly cooled to room temperature. In such conditions, the mixture must remain liquid well below its crystallization temperature, in a metastable state, giving rise to what is called supercooled liquid.^37^ It remains liquid until nucleation is mechanically activated by pouring it into a crystallizer. The first drops nucleate in contact with the crystallizer and serve as a seed to grow a solid sodium acetate monolith, as observed in Figure 4. With the thermal camera, students observe an increase of the temperature of the solid monolith during its solidification, verifying that this crystallization is an exothermic process related to the crystallization latent heat.^35^

**Figure 4:**
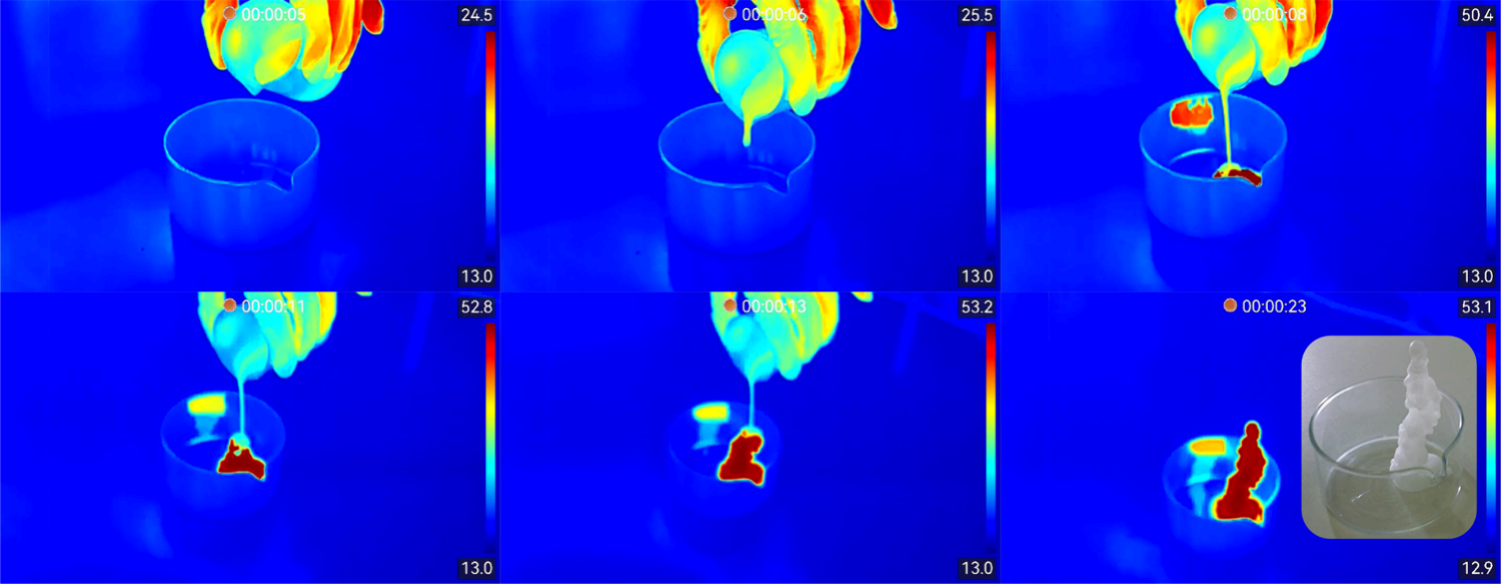
Thermal video sequence recorded with the crystallization of supercooled hydrated sodium acetate after mechanical nucleation activation by pouring it into a crystallizer, recorded with HIKMICRO SP40 in the rainbow palette. Inset: optical image of the final solid monolith.

Students have to discuss their experimental observations and the physicochemical origin of the crystallization process and draw the schematic diagram of temperature versus heat for the observed evolution of sodium acetate.

### Experiment 4: Solid-to-Liquid Phase Transitions for Passive Cooling in Thermal Energy Storage Applications

In this experiment, students delve into reversible thermal energy storage and release for both passive cooling and heating applications by preparing and observing the behavior of a composite containing a PCM. As previously mentioned in Experiment 2, passive cooling and/or heating do not require grid connection or power supply, which is of great interest for many technological applications, such as thermal management of buildings and electronic devices, among others.^38–40^

For real-world technologies, it is often difficult to work with pure PCMs with solid-to-liquid phase transitions due to leaks during melting, among other problems. One solution is the use of composites, in which the PCM is embedded in a porous solid matrix that can contain the PCM even in the liquid state. To evaluate the thermal behavior of the composite, the thermal properties of both phases (porous matrix and dispersed PCM) must be taken into account.

In this experiment, students make a phase change composite for reversible passive cooling and heating using low-cost supermarket products: a candle (as a source of paraffin wax, which acts as the PCM) and a “magic cleaning sponge” (made of microporous melamine, used as the matrix). A piece of candle is melted in a beaker using a silicone oil bath. Then, a piece of sponge is embedded by absorption in the melted candle until complete saturation. The paraffin embedded in the sponge is allowed to crystallize at room temperature. Another piece of sponge of similar size is used as a blank. The paraffin is known to present two consecutive phase transitions: a minor phase transition at *T*_s‑s_ ∼ 30–35 °C between two different solid states (solid–solid transition) and a more energetic solid-to-liquid phase transition at the melting temperature of *T*_m_ ∼ 40–50 °C.^41^

Both the blank and the composite are placed on a hot plate, ensuring good contact with the surface, and slowly heated above the melting temperature of paraffin, while the temperature evolution is monitored with a thermal camera.

During the heating stage, the thermal energy is absorbed and stored by both materials, a process known as the charging stage. Later, both materials are rapidly removed from the hot plate and placed on to a cold plate at room temperature for cooling. During this second stage, the stored thermal energy is released from the materials to the surroundings, a process known as the discharging stage.

A simple experiment can be limited just to the observation of the thermographic images and to the qualitative explanation of the temperature evolution in both samples using either the FLIR One Pro or HIKMICRO SP40 camera (Figure 5). For higher level students, they can make use of the radiometry tool of the HIKMICRO SP40 model and record the temperature evolution over time. This evolution is graphically represented in Figure 6, illustrating the charging and discharging processes.

**Figure 5:**
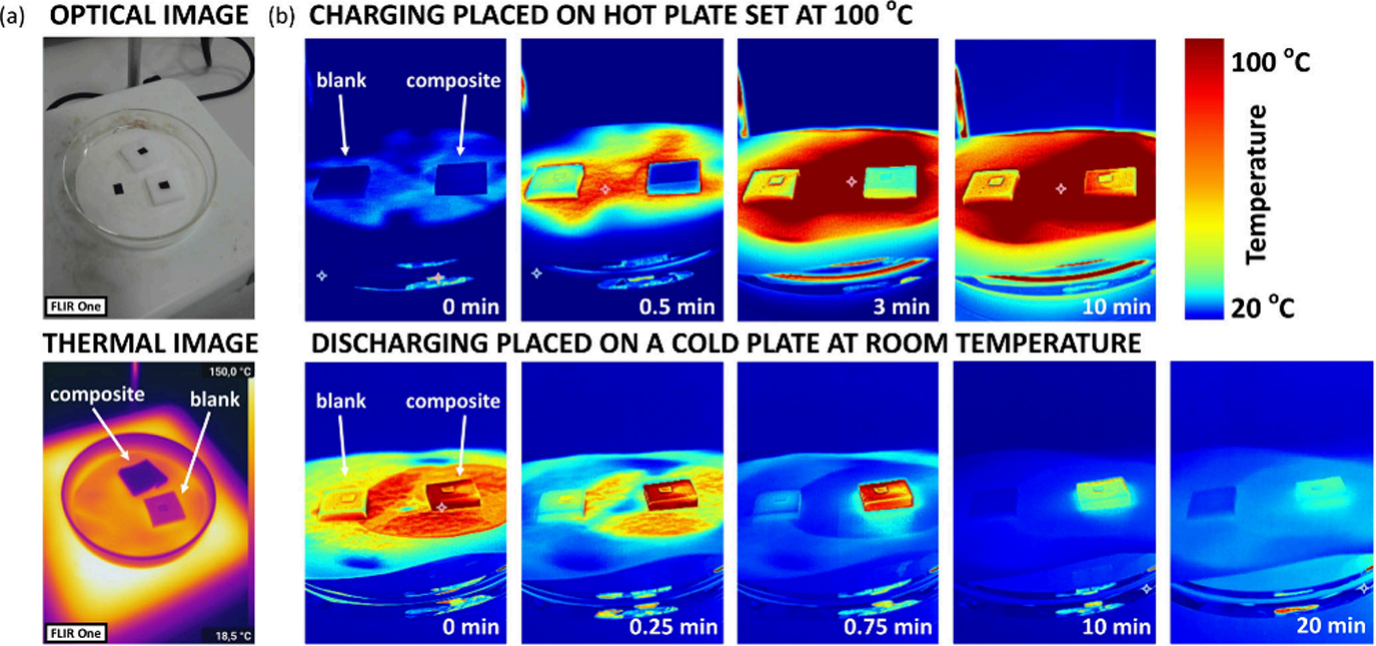
(a) Optical and thermal images recorded with a FLIR One Pro thermal camera in iron palette for the paraffin–sponge composite and for the blank sponge placed on a hot plate. (b) Thermal video sequences recorded with HIKMICRO SP40 in rainbow palette for the paraffin–sponge composite and for the blank sponge upon heating on a hot plate (charging process, top row) and upon cooling (discharging process, bottom row) when placed on a cold plate. Note: black tape is placed for temperature calibration, as explained in SI.

**Figure 6:**
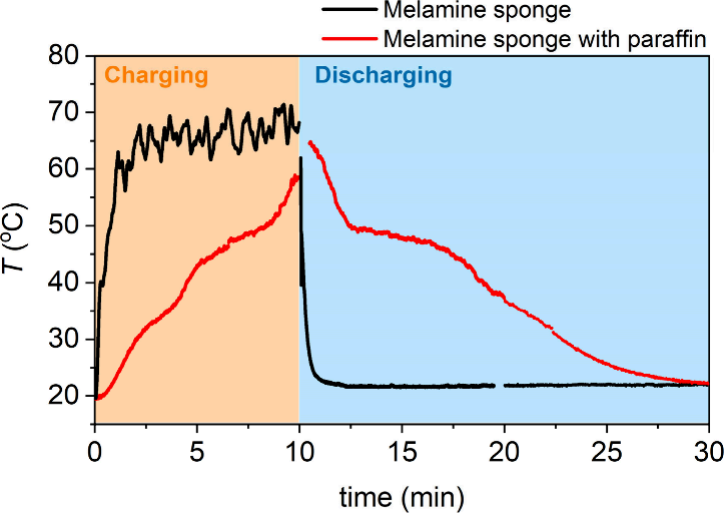
Temperature profile over time registered with the thermal camera radiometry tool for both samples, the blank melamine sponge and the paraffin–sponge composite.

In the charging stage, the blank melamine sponge rapidly reaches a maximum temperature of ∼65 °C, which is kept constant until the discharge is forced. Meanwhile, the paraffin–sponge composite increases its temperature in a more progressive manner, with several steps corresponding to the sensible heat (defined as the total thermal energy that a given material can absorb/release due to its heat capacity away from any phase transition) and the latent heat storing processes. In the same line, when the discharge is forced, by placing the materials on a cold plate, the temperature of the melamine sponge decreases to room temperature almost immediately. However, the paraffin–sponge composite releases the thermal energy at a much slower rate, again showing different steps related to both sensible and latent heat. Students should explain the observed differences based on the phase transitions of the paraffin but also consider the sensible and latent heats of both samples. In addition, the students will estimate which contribution—sensible heat or latent heat—is larger for the thermal energy storage in the case of the paraffin–melamine composite (see SI for further details).

## Conclusions

Under the growing academic interest in educational programs on energy transition, we present a new set of experiments for delving into the physicochemical foundations of phase change materials (PCMs), their thermal properties, and their applications in eco-friendly thermal energy storage, passive cooling, and heating. For that purpose, we use thermal cameras, which are becoming increasingly affordable and attractive tools for chemistry teaching. These experiments are carefully designed to interrelate key physicochemical topics that are transversal to early chemistry subjects (infrared radiation, spectroscopic transmittance, optical band gap, phase transitions), and they are applied to increase the understanding of the thermal properties of phase change materials with technological interest. Furthermore, the explanations, initially targeted at senior chemistry students, can be easily adapted to technical education, high school education, and even the general public. In turn, this work compiles an innovative set of thermography-based experiments to expand the curriculum of emerging degrees focused on chemistry-based solutions for energy transition.

## Supplementary Material





## Notes

Basic introductory information, pedagogy additional details, preliminary assessment, and recommended literature PDF, DOCX)
