# Supplementary material for: Thermal Imaging for Teaching Materials Chemistry: Phase Change Materials toward Energy Transition
Source: J Chem Educ. 2025 Jun 10;102(7):3071–7. doi: 10.1021/acs.jchemed.5c00079 (PMC12244470; doi:10.1021/acs.jchemed.5c00079)
Supplement: Supplementary file 2 [file ed5c00079_si_003.docx]

**Supplementary Information**

**Thermal Imaging for Teaching Materials Chemistry: Phase Change Materials toward Energy Transition**

Carmen de Cabo-Rodríguez, Cayetana Torremocha-Serra, Ángel Ferradanes-Martínez, María Gelpi, Pedro Dafonte-Rodríguez, Lorena Alonso-Marañón, Socorro Castro-García, Juan Manuel Bermúdez-García*.

CICA - Centro Interdisciplinar de Química e Bioloxía e Departamento Química, Facultade de Ciencias Universidade da Coruña, 15071 A Coruña, Spain.

*Email: [j.bermudez@udc.es](mailto:j.bermudez@udc.es)

**1. Basic introductory information to address at the beginning of the practical sessions:**

**1.1. Basic introduction to thermography and optical properties of materials.**

Thermography is an instrumental technique for infrared thermal imaging that registers the heat radiation (in the infrared region) emitted by the surface of different objects and generates images (thermograms) of the temperature of such objects. Here, it should be mentioned that all objects with a temperature larger than absolute zero (0 K) emit electromagnetic radiation with different wavelengths that are directly related with the temperature of the object.

In early stage academic years (first and/or second year of chemical degree), the Planck radiation law is explained to calculate the energy distribution of radiation emitted by a blackbody –an ideal body in thermal equilibrium at a given temperature that absorbs all radiant energy and re-emit this energy with an energy distribution according to equation (1):^1,2^

$E_{\lambda}=\frac{8\pi hc}{\lambda^{5}}\frac{1}{exp(hc/kT\lambda)-1}$ (1)

where *E*_λ_ is the energy density at a given wavelength of the emitted radiation, *h* is the well-known Planck’s constant, λ is the wavelength of the emitted radiation, *c* is the speed of light, and *k* is the Boltzmann constant.

In that manner, calculating the energy density of the emitted radiation at different wavelengths at a given temperature, we can obtain the so-called Planck’s radiation curves. These curves show that for any given temperature there is a wavelength where the intensity of emitted radiation is maximum, see Figure S1:


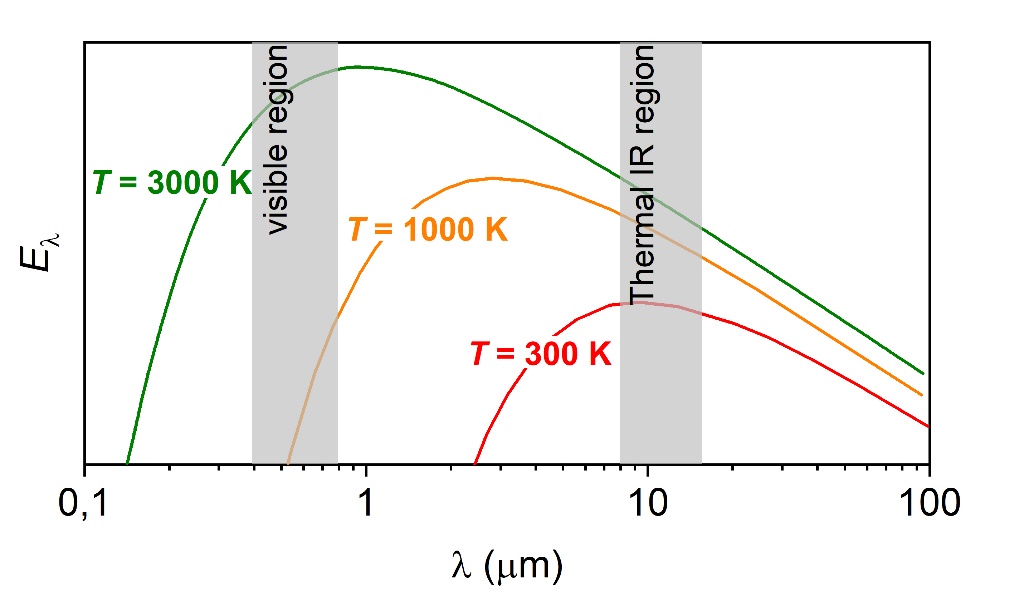


**Figure S1.** Planck’s radiation curves showing the energy density distribution of a blackbody as a function of the wavelength of the emitted radiation (*E*_λ_) at different equilibrium temperatures, namely, 3000 K, 1000 K and 300 K. Data reproduced from reference 1.

In that regard there is a simpler and previously established law –known as Wien’s displacement law– that relates the wavelength of the maximum energy density with the temperature of the object.

Wien’s law is commonly used in thermography to calculate the temperature of objects by using the wavelength of the radiation that reaches the detector of a thermal camera, according to equation (2):^1^

$T=\frac{b}{\lambda_{max}}$ (2)

where *T* is the temperature of the emitting body, *b* is Wien’s constant (~2898 μm·K), and λ_max_ is the wavelength of the maximum energy density emitted by the radiant body.

Accordingly, any object with a temperature in the range of ambient temperature will emit with maximum energy density in the infrared region of the electromagnetic spectrum.

**Effect of the atmosphere**

The emitted radiation must be transmitted through different media until reaching the thermal camera detector. In that regard, the first “obstacle” that thermal infrared radiation encounters is the atmospheric air.^1^ The chemical components of the atmosphere, especially moisture (H_2_O) and carbon dioxide (CO_2_) exhibits infrared absorption bands due to the different bonds’ vibrational modes. These concepts must have been previously studied in early academic years of the chemistry degree. Nevertheless, if we study the infrared transmission spectra of the atmosphere under defined conditions of temperature and moisture (i.e. room temperature and 50% humidity), we can observe an almost 100% transmittance window from ~8 μm to ~14 μm, which corresponds to the thermal infrared region (Figure S2). Therefore, the thermal radiation emitted by objects can travel through the atmosphere with minimal losses, within a reasonable distance between the radiant object and the thermal camera.^1^


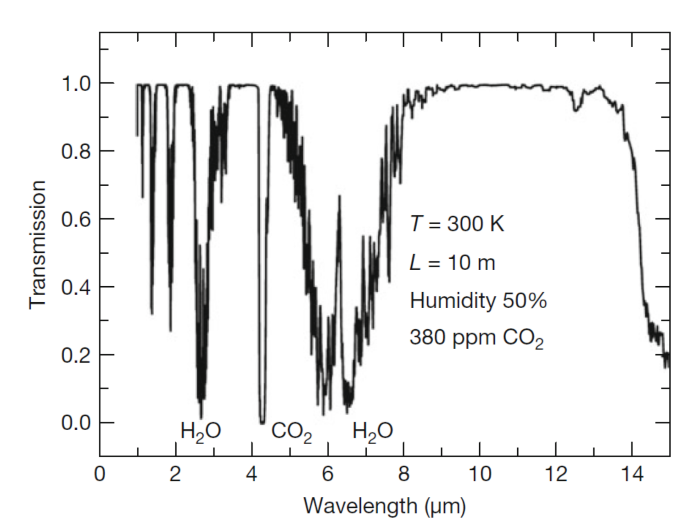


**Figure S2.** Transmission spectra for the atmosphere in conditions of room temperature and 50% of relative humidity. Infrared absorption bands for H_2_O and CO_2_ are identified in the spectrum. Figure reproduced with permission from reference 1.

**Calibration of the thermal camera**

To calibrate the thermal camera for accurate quantitative analysis, it is necessary to know the thermal radiation emission efficiency of the material to be measured, in other words, its emissivity. Another option is to place a black tape, which will function as a black body with a known emissivity of ~1 (close to 100% emission efficiency), on the surface of the material, and wait until the tape and the material are in thermal equilibrium. ^1^

**Optical properties of the materials**

Depending on the chemical nature of the material, this material will exhibit absorption bands in different regions of the electromagnetic spectrum.

These optical properties can be explained in a simple manner in terms of the electronic band structure and the lattice vibrations of the materials.

According to the band theory, all solid materials (crystalline and amorphous) can exhibit an electronic band structure formed by a linear combination of atomic orbitals (LCAO) of the *N* atoms forming the solid. These concepts must have been studied in early (first and/or second) academic years of the chemistry degree.^2,4^

In metals, the band is continuous overlapping electron-filled lower energy levels with empty higher energy levels (see Figure S3). However, in semiconductors and insulators, there is an energy gap between the lower energy band (valence band formed by LCAO of bonding atomic orbitals) and the higher energy band (conduction band formed by LCAO of anti-bonding atomic orbitals), see Figure S3. This energy gap, known as band gap or *E*_g_, is a discontinuity in the electronic bands of the solid materials that arise from forbidden energy levels that cannot be occupied by electrons. Accordingly, from an electron to be promoted from the valence band to the conduction band must gain an energy equivalent and/or superior to that of the band gap.^2,4^

This band gap directly affects to the optical properties of the materials. In that manner, most insulators –crystalline (such as Al_2_O_3_) or amorphous (such as SiO_2_ glasses)– tend to be colourless and transparent to the visible radiation (note that colouration in insulator are due to the presence of impurities, which is out of the scope of this introductory explanation).^2^ The reason for this transparency lie in the noticeably large band gap, where electrons must absorb very energetic radiation in order to be promoted from the valence band to the conduction band. Actually, this energetic radiation generally corresponds to ultraviolet light.^2^


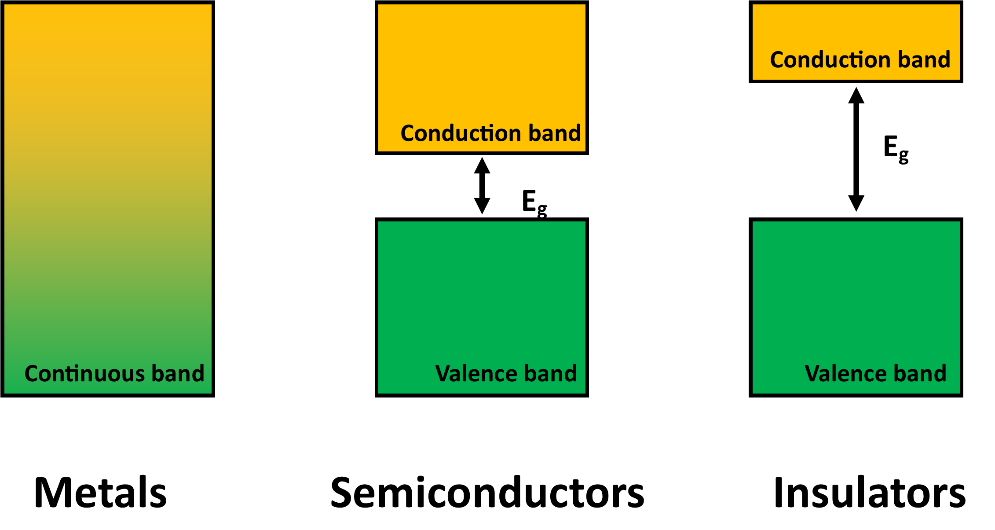


**Figure S3.** Schematic representation of electronic bands for metals, semiconductors and insulators.

Meanwhile, semiconductors present significantly lower band gaps with energy in the range of visible and/or even near infrared region of the electromagnetic spectrum. That is the case of ZnSe and Ge, respectively.^2^ Therefore, all insulators and semiconductors will absorb radiation of different wavelength related to the energy of their band gap to promote electrons from the valence to the conduction band.^2^

In addition, all solids present lattice vibrations in a similar way that the bonds vibrations of molecular species. These lattice vibrations behave as mechanical oscillators that can absorb radiation, giving rise to vibrational transitions, although with much lower energy than in the case of interband electronic transitions. Accordingly, depending on the chemical nature of the lattice bonds, there will be materials that will absorb energy in the thermal infrared region while others will be transparent to this radiation.^5^

**1.2. Basic introduction to thermal properties of phase change materials in the context of refrigeration, heating and thermal energy storage:**

In a broad definition, a phase change material (PCM) is any material that can transform from one phase to another when exposed to an external stimulus, such as temperature. These phase changes can occur within two different aggregation states (i.e. solid-liquid, liquid-gas) or even within the same aggregation state (i.e. solid-solid).^6,7^ Some PCMs exhibit phase transitions at temperatures of interest for technological applications and can be reversibly driven, so energy can be stored during the forward transition (i.e. solid to liquid –or melting) and released during the backward transition (i.e. liquid to solid –or crystallization).^6^

This is the basic principle of thermal energy storage in buildings, where a PCM is used to absorb and store the thermal energy of sunlight during the day, avoiding the overheating of the inner spaces. Meanwhile, when the ambient temperature decrease at night, the stored energy is released, so energy consumption for space heating is minimized.

This practical phenomenon can be related to the temperature *versus* heat diagrams commonly used to explain the thermal behaviour of a material during a phase transition (Figure S4).^6,7^


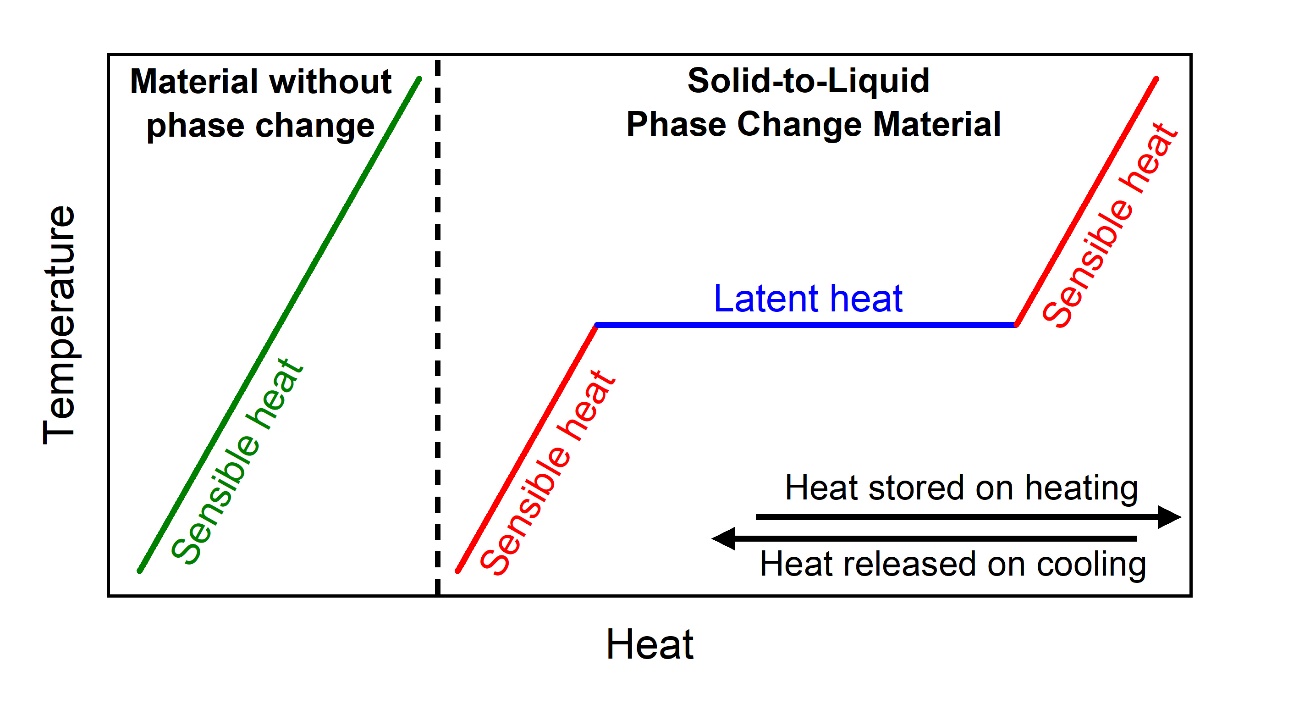


**Figure S4.** Schematic temperature *versus* heat diagram showing the sensible and/or latent heat for a material without phase change (left) and for a solid-to-liquid phase change material (right). Note: Black arrows indicate the process direction for thermal energy storage and release on heating and cooling, respectively. Figure adapted from reference 6.

When a phase change material is heated using an external heat source (for instance sunlight or residual heat from an engine or a chemical reactor), the added thermal energy increases the temperature of the material until it reaches the transition temperature, following equation (3):^6,7^

$q_{heating}=m\times C_{p}\times\Delta T$ (3)

where *q*_heating_ is the added thermal energy used to heat up the material, *m* is the sample mass, *C*_p_ is the specific heat (that will be explained later), and ΔT is the temperature change during the material heating.

Then, during the phase transition, the material stays under an isothermal stage (at constant temperature) while the added thermal energy is used to induce such phase change, following equation (4):^6,7^

$q_{transition}=m\times{\Delta H}_{transition}$ (4)

where *q*_transition_ is the thermal energy absorbed during the phase transition, m is the sample mass, and Δ*H*_transition_ is the enthalpy change of the transition or specific latent heat (which will be later defined).

These two thermal phenomena –described by equation (3) and (4)– already highlight two important thermal properties of phase change materials, namely the heat capacity and the latent heat.

The **specific heat capacity** can be understood as the amount of thermal energy that can be stored in a material per mass unit provoking a temperature increase away from a phase transition.

This specific heat capacity is the responsible for thermal energy storage in a material in the form known as **sensible heat** (total thermal energy absorbed and stored for a given mass of material when warming it up away from the phase transition, equivalent to *q*_heating_ in equation (3)). For instance, the **sensible heat** of water is used in hot-water bags for warming our beds and/or injured joints.

Meanwhile, the **specific latent heat** can be understood as the amount of thermal energy that can be stored during a phase transition of a material per unit of mass. Here, the specific latent heat is responsible for the thermal energy storage in a material in the form of **latent heat** (total thermal energy absorbed and stored for a given mass of material during the phase transition, equivalent to *q*_transition_ in equation (4)). This is commonly found in the “click” or “instant” heat-packs or hand-warmers, where a liquid-to-solid transition releases a large amount of thermal energy.

Accordingly, a phase change material can store thermal energy in the form of both **sensible heat** (which is related to the specific heat capacity) and **latent heat** (related to the enthalpy change of the transition, also known as the specific latent heat).

In general terms, sensible heat is noticeably lower than latent heat. Therefore, latent heat storage is more adequate and efficient for thermal energy storage applications, see Figure S4.^6,7^

Although temperature changes are not observed during phase transitions induced by external heating, it is possible to observe temperature changes associated to phase transitions under quasi-adiabatic conditions, such as fast crystallization or evaporation.

In that regard, thermography can be used to observed those fast temperature changes and relate them to the enthalpy change of the transition and the specific heat capacity of the material, according to the first law of thermodynamics and using equation (5):^6,7^

${\Delta H}_{transition}=C_{p}\times\Delta T$ (5)

Although this method presents some limitations (different values of *C*_p_ related to the coexistence of different phases in equilibrium during the phase transition, partial thermal energy exchange from the PCMs to the surroundings, etc.), the calculated values would be close enough for a demonstration session and serve as a practical illustration for explaining theoretical thermal concepts generally used in the field of phase change materials and thermal energy storage.

In the same line, there is another important thermal parameter yet to be considered for thermal energy storage and release applications: the thermal conductivity.

**2. Pedagogy details of “Experiment 1: Understanding the Basics of Thermal Imaging”.**

**2.1. Pedagogy objectives:**

- Introduction to the use of thermography as a characterization technique for thermal properties of materials.

- Understanding the relation of thermal infrared radiation with the Planck’s radiation law and with the Wien’s displacement law.

- Establishing relationships between the optical properties of materials, the infrared spectrum and thermography.

- Deepening into physicochemical concepts applied to thermography: optical properties, transmittance windows, semiconductors, and optical band-gap.

**2.2. Time distribution.**

This experiment is designed for **a 60 minutes session**:

- **25 minutes** of introduction to thermography, and hands-on manipulation of thermal cameras in order to get used to the software, temperature scale, focal distance, and colour palettes.
- **35 minutes** of taking thermal and optical images of an Erlenmeyer flask filled with hot water placing lenses of different materials between the Erlenmeyer flask and the thermal camera.

**2.3.** **Questions to be addressed by the students:**

- Calculate the wavelength of the thermal radiation emitted by the Erlenmeyer flask, using the Wiens’ displacement law.

- Explain the experimental observation of the optical and thermal images through the different lenses on the basis of optical properties, optical band-gap and electronic band theory and lattice vibrations of the different materials.

- Why the thermal camera registered the Erlenmeyer flask as “hot” if it is made of borosilicate glass?

**3. Pedagogy details of “Experiment 2: Liquid-to-Gas Phase Transitions for Evaporative Cooling”, and “Experiment 3: Liquid-to-Solid Phase Transitions for Heating in Thermal Energy Storage Applications”.**

**3.1. Pedagogy objectives:**

- Introduction to thermal properties of phase transitions, passive cooling and thermal energy storage.

- Studying thermal properties of PCMs under quasi-adiabatic conditions.

- Performing quantitative temperature readings using thermal cameras.

- Understanding sensible heat and latent heat diagrams.

- Calculating temperature change using specific heat capacity and specific latent heat.

**3.2. Time distribution.**

Experiments 2 and 3 are designed for two **combined sessions of 60 minutes each to be performed in different days:**

**First session:**

- **15 minutes** for setting the ceramic pot *set-up* (filling one pot with water at ambient temperature) and taking the initial temperature readings with the thermal camera. The temperature will be measured again at the end of the session.
- **15 minutes** for preparing the hydrated sodium acetate PCM.
- **30 minutes** of introduction to thermal properties of phase change materials in the context of refrigeration, heating and thermal energy storage.

**Second session (next day):**

- **15 minutes** to record the temperature of the ceramic pot and compare with the previous measurements.
- **15 minutes** to trigger the crystallization of hydrated sodium acetate and record the temperature evolution with the thermal camera.
- **30 minutes** of internal discussions and questions solving.

**3.3.** **Questions to be addressed by the students:**

- Explain the endothermic and exothermic processes (from a chemical point of view) observed in the ceramic pot and in the sodium acetate experiments.

- Calculate the temperature change that can be obtained in 1 L of water with the evaporation of 20 g of such water. (*C*_p_ ~ 4.2 kJ K^-1^ kg^-1^, Δ*H* ~ 2430 kJ Kg^-1^).

- Draw a schematic temperature *versus* heat diagram (similar to Figure S1) for the hydrated sodium acetate during the stages of melting, subcooling and crystallization attending to Figure S1. Further details for the diagrams construction can be found in reference. ^9^

**4. Pedagogy details of “Experiment 4: Solid-to-Liquid Phase Transitions for Passive Cooling in Thermal Energy Storage Application”.**

The observed thermal response in a real material cannot be often attributed to one simple thermal phenomena, for instance just to the latent heat of a single phase transition. Moreover, for practical applications, a process must present reversibility over time. Therefore, it is important for the students to develop a critical thinking and identify all the possible contributions to an experimentally observed phenomenon as well as reversible processes. Those are of the main objectives of experiment 4 as listed below.

**4.1. Pedagogy objectives:**

- Introduction to composite thermal materials and their potential applications for reversible passive cooling and passive heating.

- Further deepening into thermography experiments from the previous qualitative and/or semi-quantitative experiments into continuous quantitative experiments using the radiometry tool of the thermal camera.

- Learning about thermography calibration methods using a blackbody reference for correct quantitative analysis.

- Integrating all the knowledge acquired during the introductory theoretical sessions and the previous experiments to identify all possible contributions to the experimentally observed thermal behaviour of a composite embedded with a phase transition material.

**4.2. Time distribution.**

This experiment is designed for **a 60 minutes session**:

- **20 minutes** of composite preparation by identifying the solid-liquid transition temperature of paraffin, melting the paraffin in a beaker and embedding a piece of melamine foam in it.
- **40 minutes** of recording a thermal video using the radiometric quantitative tool of the thermal camera, and parallel internal discussions.

**4.3.** **Questions to be addressed by the students:**

- Identify the different processes taking place during the charging and discharging stages of the paraffin-sponge composite (sensible heat, latent heat, solid-solid phase transition, solid-liquid phase transition).

- Attending to the time-length of the solid-solid and solid-liquid temperature septs, which one would be more energetic?

- Why the temperature of the blank sponge increases faster than that of the paraffin-melamine composite? Which thermal properties are contributing to these response?

- Calculate the total thermal energy stored when heating this composite from 20^o^C to 60^o^C. Consider 95%wt of paraffin and 5%wt of melamine sponge, *m*_paraffin_ = 50 g, C_p(solid paraffin)_ ~ 2.1 kJ K^-1^ kg^-1^, C_p(melted paraffin)_ ~ 2.9 kJ K^-1^ kg^-1^, *C*_p(melamine foam)_~ 1.2 kJ K^-1^ kg^-1^, Δ*H*_solid-solid_ ~ 35.0 kJ kg^-1^, *T*_s-s_ ~ 35^o^C, Δ*H*_solid-liquid_ ~ 150.0 kJ kg^-1^, *T*_m_ ~ 55^o^C.

Which contribution would be larger: sensible heat, latent heat from the solid-solid phase transition or latent heat from the solid-liquid phase transition?

**5. Preliminary evaluation of the use of thermal imaging for education of phase change materials.**

In order to perform a preliminary evaluation of thermal imaging for education of phase change materials, we have adapted the proposed experiments for a 2-hours hands-on sessions for university students of the Master in Sciences, Technologies and Environmental Management (6 students), and for non-university students from the Higher Technical Education in Laboratory Analyses (16 students), equivalent to the Technical College in USA or the Higher National Diploma in UK. The selected students were previously trained in calorimetry analysis, so they were able to compare this technique with the proposed thermal imaging, and additional thermal analysis instruments and demonstrators.

In the first hour of the session, students are provided with sensorial demonstrators (commercial hand-warmers of hydrated sodium acetate, and compressed-air cans), so they can feel exothermic and endothermic processes related to the phase transformation from the liquid-to-solid phase transition in the hand warmers, and from the liquid-to-gas transition in the air-compressed can. In addition, they use thermal cameras, temperature-probes coupled with data-loggers, and infrared temperature guns to observe the thermal changes of the hand-warmers, the air-compressed cans and also from the ceramic pots explained in experiment 2. During the second hour of the session, the students observe the temperature changes of a melamine blank sponge and a sponge-paraffin composite (from experiment 4) upon heating and cooling using both a thermal camera and a temperature-probe coupled with data-logger.

After this session, we evaluate the level of engagement and attraction of these techniques for the students, the ease of manipulation and of data interpretation, and the amount of the relevant thermal information that these techniques can provide for the students.


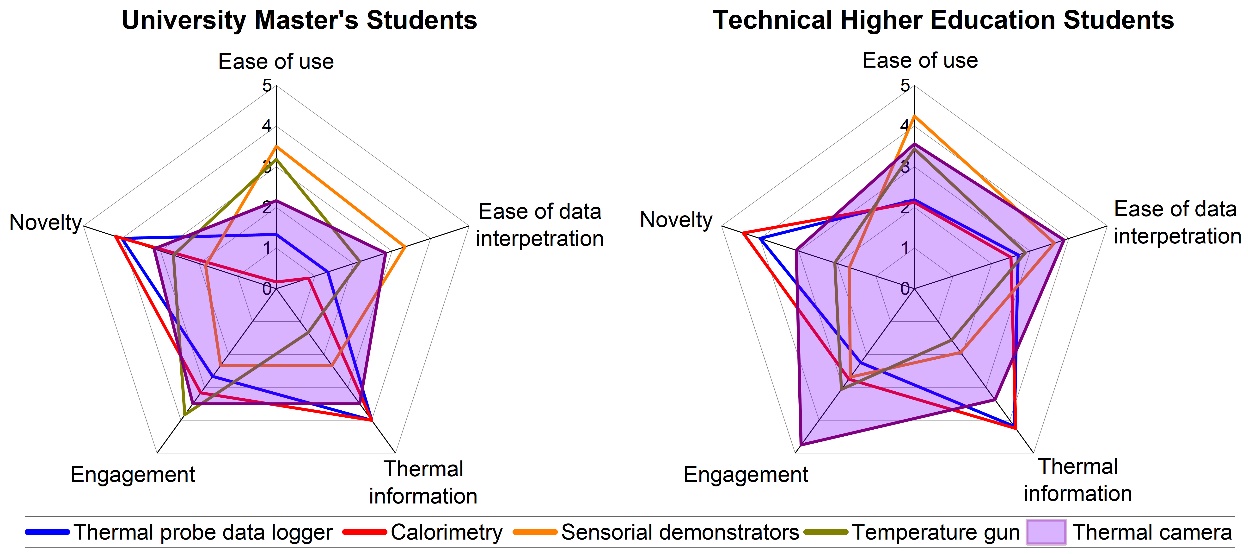


**Figure S5.** Charts comparing the perception of university master’s students (n = 6) and technical higher education students (n = 16) regarding the ease of use and data interpretation, amount of relevant thermal information, engagement and novelty of different thermal analysis instruments and sensorial demonstrators.

Considering the students’ perceptions in this preliminary assessment, Figure S5 shows this evaluation for the different aspects in a scale from 1 to 5 (lower to higher). Remarkably, thermal cameras show the best balance between all these characteristics, while calorimetry and thermal probes offer less engagement and more difficulty in both instrument manipulation and data interpretation.

**6. Recommended literature:**

1 M. Vollmer and K.-P. Möllmann, *Infrared Thermal Imaging: Fundamentals, Research and Applications*, Wiley, 2nd edn., **2017**.

2 M. Fox, *Optical Properties of Solids*, Oxford University Press, Oxford, **2001**.

3 R. E. Fischer, B. Tadic-Galeb and P. R. Yoder, *Optical System Design*, McGrawHill, 2nd edn., **2008**.

4 A. R. West, *Solid state chemistry and its applications.*, John Wiley & sons Ltd., 2nd edn., **2014**.

5 S. Nudelman and S. S. Mitra, in *Optical Properties of Solids. Optical Physics and Engineering.*, Springer, **1969**.

6 H. M. Ali, *Advanced Materials-Based Thermally Enhanced Phase Change Materials*, Elsevier, **2024**.

7 J. Khachan, *Thermal Properties of Matter*, **2018**.

8 Engineering Tool Box Website, https://www.engineeringtoolbox.com/.

9 J. Lizana, P. E. Sanchez-Jimenez, R. Chacartegui, J. A. Becerra and L. A. Perez-Maqueda, *J. Energy Storage*, **2022**, 55, 105584.
